# Phosphorylated chitosan accelerates dermal wound healing in diabetic wistar rats

**DOI:** 10.1007/s10719-022-10093-5

**Published:** 2022-11-30

**Authors:** U. Anushree, Pratik Punj, Vasumathi, Sanjay Bharati

**Affiliations:** Department of Nuclear Medicine, Manipal College of Health Professions, Manipal Academy of Higher Education, Manipal, 576104 Karnataka India

**Keywords:** Diabetes mellitus, Chitosan, Phosphorylation, Granulation tissue, Excisional wound

## Abstract

Phosphorylated chitosan (PC), a water-soluble derivative of chitosan possesses several biological and chemical properties suitable for diabetic wound healing. In the present study, we report the synthesis and diabetic wound healing capabilities of PC. Elemental analysis, FT-IR, ^13^C-NMR and ^31^P-NMR techniques were employed for the chemical characterization of PC. *In vitro*, antioxidant properties of PC were determined in terms of Fe^3+^ reducing, metal chelating, lipid peroxidation and superoxide scavenging ability. The wound healing potential of PC was assessed in diabetic excisional wound rat model. PC exhibited good water solubility, and *in vitro* antioxidant capacity. Wound contraction was higher in PC-treated wounds (91.11%) as compared to untreated wounds (67.26%) on 14^th^-day post wound creation. Histopathology of PC-treated wounds revealed improved tissue morphology with higher number of fibroblasts, a thicker epithelial layer, enhanced collagen deposits and angiogenesis as compared to untreated wounds. An overall increase of 57% and 25% in hydroxylamine and hexosamine content respectively were noted as compared to untreated wounds. A significant (*P* ≤ 0.05) increase in SOD activity and a significant (*P* ≤ 0.05) decrease in lipid peroxides were recorded in PC-treated wounds as compared to untreated wounds. These observations demonstrated that PC can be used as an effective agent in diabetic wound healing.

**Graphical Abstract:**

Illustration of phosphorylated chitosan (PC) synthesis and its wound healing potential: Chitosan was phosphorylated to impart diabetic wound healing properties. Chemical characterizations such as elemental analysis, FT-IR and NMR confirmed successful phosphorylation of chitosan. PC exhibited good *in vitro* antioxidant properties. To assess the diabetic wound healing potential, an excisional wound model was developed in diabetic rats. PC treatment demonstrated accelerated wound healing.

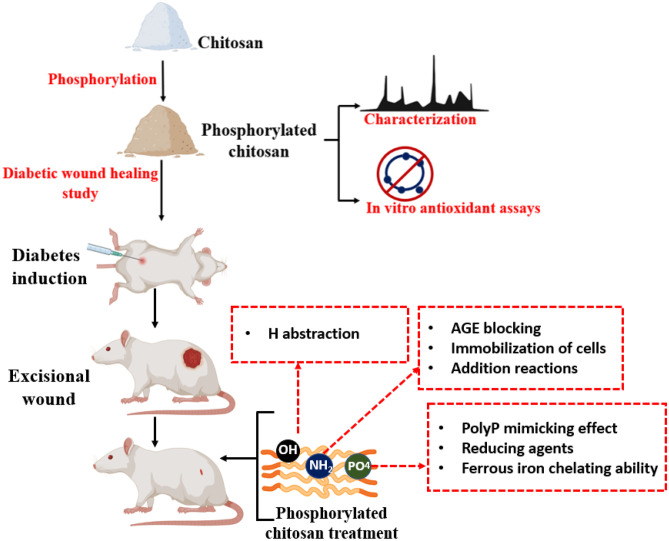

**Supplementary Information:**

The online version contains supplementary material available at 10.1007/s10719-022-10093-5.

## Introduction

Wound healing is an essential biological process consisting of an intricate sequence of cellular and biochemical responses [1]. The process of wound healing can be divided into four overlapping phases: hemostatic, inflammatory, proliferative and remodeling or maturation phase [2]. The hemostatic phase is marked by immediate response of the body to prevent blood loss through vasoconstriction and formation of a blood clot. The inflammatory stage is mainly associated with the prevention of infection via infiltration of neutrophils, macrophages, and lymphocytes. Once the inflammatory system is stabilized and the site is free of debris, the proliferative phase begins to repair the defective wound via the formation of granulation tissue, angiogenesis, collagen deposition, and wound contraction simultaneously. In the last phase, the tissue structure is regained through rearrangement of collagen, development of normal epithelium and scar tissue maturation [3, 4].

In contrast, the wound healing in diabetes mellitus is derailed and associated with a chronic inflammatory phase, reduced neovascularization, increased oxidative stress, and impaired collagen deposition [5]. The hyperglycaemic conditions in diabetes mellitus result in the glycation of key functional proteins as well as cell membrane phospholipids forming advanced glycation end products (AGE) at the wound site [6, 7]. These AGEs are documented to interfere in wound healing either directly or through receptors corresponding to AGEs (RAGEs) [7]. The accumulation and/or interactions of these AGEs are known to impede the transendothelial migration of inflammatory cells thereby prolonging inflammatory response [8]. Furthermore, AGEs present in the vascular membranes of wounded tissue delay the recruitment of endothelial progenitor cells required for angiogenesis. These events are further accompanied by structural changes in the fibroblast membranes leading to premature apoptosis, reduced collagen synthesis, and deposition [7]. Collectively, all these processes result in the failure of diabetic wound contraction and closure.

Phosphorylated chitosan (PC), a water-soluble derivative of chitosan possess several favorable wound healing properties such as hemostatic properties, metal chelating ability, antioxidant, anti-inflammatory, bactericidal and angiogenic activity. Additionally, PC has the potential to modulate the behavior of fibroblasts and endothelial cells due to the presence of anionic groups [9, 10]. The amphoteric nature of PC provides flexibility to the cells to adhere and proliferate in the extracellular matrix (ECM) [10]. Furthermore, it has the potential to immobilize various growth factors such as fibroblast growth factor-2 (FGF2), vascular endothelial growth factor (VEGF), and epidermal growth factors (EGF) present in the vicinity of the cells [11]. Hence, the cell surface-based interaction of these growth factors will then lead to growth, secretion, and thereby subsequent regeneration of connective tissues [12]. Considering these properties of PC, we hypothesize that PC may be effective for diabetic wound healing.

## Materials and methods

### Chemicals and kits

Low molecular weight chitosan (CAS: 9012–76-4) and streptozotocin (CAS: 18883–66-4) were procured from Sigma Aldrich Co. (St. Louis, USA). The average molecular weight of chitosan used in the study was further established by intrinsic viscosity method using the Mark-Hauwink equation [13] and found to be 134 kDa. The degree of deacetylation of chitosan was determined to be 72% using FTIR spectroscopy [14]. Orthophosphoric acid (85%) and urea were procured from Sisco Research Laboratories Pvt. Ltd. (Mumbai, India). Masson’s trichrome staining kit for collagen content estimation was supplied by AMD labs (Bangalore, India). Other chemicals used in the study were of prime quality and obtained from reputed national and international firms.

### Synthesis of phosphorylated chitosan

Chitosan was phosphorylated by the dimethyl formamide method as described by Jayakumar et al. with several modifications [15]. Briefly, chitosan (0.25 g) was dissolved in dimethyl formamide (20 mL). 3.75 g of urea and 3 mL of orthophosphoric acid were added and heated at 150 ºC for 30 min. The precipitate obtained was washed thoroughly in methanol and dissolved in double distilled water (DDW). The pH was adjusted to 10.5 using NaOH (1 N). The obtained compound was dialyzed against the DDW using a 12,000 Da cut-off membrane for 48 h. The final phosphorylated chitosan (PC) solution was lyophilized for further use. The purity of the compound was determined by thin layer chromatography. 3 µL (5 mg/mL) of PC and chitosan in 50% of methanol respectively was spotted on TLC plates ( Merck 60 F 254), with a mixture of butanol, acetic acid, and water as mobile phase (5:1.5:3.5) at room temperature. Visualization of the compound was obtained by 0.2% of ninhydrin in ethanol and heating at 110 °C.

### Solubility test of PC

Solubility of PC was determined (2 mg/ mL) in acetic acid (0.1 M, pH 3.4), sodium hydrogen carbonate (0.1 M, pH 8.6), and DDW (pH 6.6) at room temperature according to the method described earlier [16]. The solutions were stirred for 3 h and filtered (0.45 µm). Solubility of PC was determined as a change in filter paper weight and evaluated as percent soluble PC compared to the total weight of PC.

### Characterization of PC

Elemental composition of PC was analyzed using energy dispersive X-ray spectroscopy (Electron dispersive X-ray spectroscope (SEM), OXFORD XMXN, Abingdon, United Kingdom). For the measurements, lyophilized samples were mounted on the sample holder using double-sided conductive carbon tapes. The samples were then coated with Au sputtering coat for surface analysis and elemental composition. The percentage of phosphorous obtained (weight% of PC) was then used to determine the degree of substitution. Results were presented as Mean ± SD.

The information regarding molecular bonds of PC and chitosan was obtained from FT-IR spectra. The Lyophilized samples were read using an FT-IR spectrometer (FTIR- 8300, Shimadzu, Japan) in the frequency range of 4000- 500 cm^−1^. The data obtained was baselined, normalized, and investigated for the incorporation of phosphate groups and bonds onto chitosan.

^13^C-NMR and ^31^P-NMR of PC were performed to determine the type/position of carbons and phosphate respectively. The lyophilized sample was dissolved in D_2_O and read at 400 MHz using high resolution multinuclear FT-NMR spectrometer (AV400 Bruker, US). The obtained spectrum were analyzed for the addition of peaks pertaining to the modification of carbons and introduction of phosphate in PC.

The number average molecular weight (M_n_) of PC was determined by reduced osmotic pressure extrapolated to zero of various concentrations of PC [17]. Briefly, osmotic pressure ($\pi$) of different concentrations (C) of PC (1–4%) was determined using an osmometer (Löser Messtechnik, Berlin, Germany). The number average molecular weight (Mn) was obtained using an equation 

$$\frac{\pi }{C}=\frac{RT}{M}$$

where, R = 0.08206 L atm mol^−1^ K^−1^, T = 298.15 K and M = Molecular weight of the compound (g/mol).

### *In vitro* antioxidant assays for PC

#### Reducing power

Ferric ion reducing antioxidant power (FRAP) of PC was assessed by the method of Xing et al. with slight modifications [18]. Briefly, 1 mL of different dilutions of PC (0.25, 0.5 and 1 mg) in phosphate buffer (0.2 M, pH 6.6) were treated with potassium ferricyanide (1% w/v) at 50 °C for 20 min. The reaction was stopped by the addition of trichloro acetic acid (10%, w/v) and centrifuged at 1000 × g for 10 min. The supernatant was mixed with ferric chloride (0.1% w/v) and incubated at room temperature for 30 min. The absorbance was read at 700 nm and a higher value of absorbance indicated increased power of reduction. The absorbance of test was compared with ascorbic acid taken as the gold standard.

#### Metal ion chelating assay

The metal chelating potential of PC was determined according to the procedure described by Ruiz-Navajas et al. with some modifications [19]. Briefly, the reaction solution containing different concentrations of PC (0.25, 0.5, and 1 mg), ferrous chloride (2 mM), and DDW were incubated for 5 min. The reaction was initiated by the addition of ferrozine (5 mM). The solution was mixed vigorously and incubated for 10 min. The decreased formation of the Fe^2+^-ferrozine complex in the presence of PC was determined from the absorbance at 562 nm. The metal chelating potential of the compound was evaluated as

$$chelating\;effect\;\left(\%\right)=(1-\frac{Absorbance\;of\;the\;sample}{Absorbance\;of\;the\;control})\times100$$

In this experiment, EDTA was kept as gold standard.

#### Superoxide radical scavenging activity

The superoxide radical scavenging ability of PC was determined as described by Feng et al. [20]. The reaction mixture contained 3 mM methionine, 2 µM riboflavin, 100 µM EDTA, 75 µM NBT, 50 mM sodium phosphate buffer (pH 7.8) and PC (different concentrations). The formation of blue formazan was measured as an increase in absorbance at 560 nm after 10 min of exposure to a light source from a fluorescent lamp. The percent inhibition was calculated as

$$\%\;inhibition=\frac{(Ao-A1)}{Ao}\times100$$

where Ao represents absorbance of blank (kept in dark) and A1 represents absorbance of the sample.

#### Lipid peroxidation (LPO) assay

Thiobarbituric acid reactive substances assay was used to measure the potential of PC to inhibit lipid peroxidation [21]. Briefly, 0.5 mL of egg homogenates (10% v/v) were mixed with 0.1 mL of different concentrations of PC (0.25, 0.5 and 1 mg/ mL). 0.05 mL of ferrous sulfate (0.07 M) was added to each tube to induce the peroxidation of lipids and kept at room temperature for 30 min followed by the addition of 1.5 mL of acetic acid (pH 3.5, 20% v/v) and 1.5 mL thiobarbituric acid (0.8% w/v in sodium dodecyl sulfate). The mixture was incubated at 95 °C for 60 min followed by the addition of butanol (5 mL) and centrifuged (1000 × g) for 10 min. The organic upper layer was decanted and read at 532 nm. The lipid peroxidation inhibitory activity of PC was expressed as a percentage and calculated as

$$\%\;inhibition=(1-\frac EC)\times100$$

where C is the absorbance of the control with egg yolk homogenate, and E is the absorbance of the test sample.

### *In vivo* assessment of PC

All the experimental procedures were approved by the institutional animal ethics committee and were conducted according to the committee for the purpose of control and supervision of experiments on animals guidelines (Reference number: IAEC/ KMC/119/ 2020). Male wistar rats (*n* = 15) were housed in standard temperature (25 ± 2 °C), humidity (65–80%), and light and dark cycle (12:12). During the entire study, period animals were fed a normal pellet diet and water *ad libitum*. The animals were acclimatized to the conditions (1 week) prior to the start of the experiments.

#### *In vivo* diabetic wound healing capabilities of PC

Diabetes mellitus was induced according to the method of Bhat et al. with minor modifications [22]. Briefly, prior to the induction of diabetes, rats were kept on fasting for 12 h followed by a single dose intraperitoneal injection of streptozotocin (50 mg/kg BW dissolved in 0.1 M cold citric acid buffer (pH 4.5). After 1 week of the stabilization period, on 8^th^ day the onset of diabetes was confirmed by estimating the fasting blood glucose levels via tail vein using a glucometer (Accu-check Performa, Roche Diabetes Care India Pvt. Ltd.). The rats with fasting blood glucose $\ge$ 300 mg/ dL were included in the experiment.

#### Animal grouping and creation of wound

The rats were divided into 3 groups (each *n* = 5), viz; normal wound, diabetic wound and diabetic wound + PC. Wounds were created in all animals according to the method described by Masood et al. with few modifications [23]. The dorsal skin surface of animals was shaved and sterilized using ethanol. The animals were then anesthetized, and the area of the skin was marked with a template (2 cm^2^ area) by methylene blue. A full thickness wound was created by cutting out a piece of marked skin extending up to the muscle layer. After wound creation, each animal was placed in a separate cage with a basement metallic tray. Wounds in the diabetic wound + PC group were treated with PC (10% in PBS, 100 µL soaked in the sterile gauze). Wounds in the normal wound and diabetic wound groups were treated with PBS (100 µL, soaked in the sterile gauze). After the application of respective treatment, the wounds were covered with the restraining bandage to fix the wound dressing. All the wounds were redressed with respective treatment on 3^rd^, 6^th^, 9^th^ and 12^th^-day post-wound creation.

#### Evaluation of wound healing

The wound healing in terms of appearance and closure of wounds were assessed from the photographs of the wound area taken using a digital camera (Canon EOS 3000D, 18MP) on the 0^th^, 3^rd^, 6^th^, 9^th^, 12^th^, and 14^th^ -day post-wound creation. The area of wound contraction was determined from peripheral wound outlines drawn on transparent sheets at all assessment time points [24]. The wound contraction (%) was calculated as:

$$=\frac{wound\;area\;on\;day\;0-wound\;area\;on\;day\;(n)}{wound\;area\;on\;day\;0}\times100$$

#### Histopathological analysis

The wound tissue was processed for Haematoxylin and eosin (H & E) staining as described earlier [25]. In brief, the excised wound tissue from all three groups was fixed in 10% formalin. After 24 h, the tissues were dehydrated in ascending grades of alcohol, cleared in xylene, and embedded in paraffin wax. The 5 µm thin tissue sections were obtained using microtome (Leica Biosystems,Wetzlar, Germany) and stained with H & E. The stained slides were viewed under the light microscope (Magnus MLX plus, India).

#### Estimation of collagen content

##### Hydroxyproline and hexosamine assays

The hydroxyproline and hexosamine content of regenerated wound tissue was determined on the 14^th^-day post wound creation. Prior to the estimations, tissues were dried in a hot-air oven at 70 °C, weighed, treated with HCl (6 N), and hydrolyzed for 4 h at 130 °C. Further, the acid hydrolysate was neutralized to pH 7.0 using 4 N NaOH. Following this, hydroxyproline content was analyzed in the hydrolysate as described by Woessner [26]. Briefly, the reaction mixture contained acid hydrolysate, chloramine T (0.05 M), perchloric acid (3 M), and p-dimethylamino benzaldehyde (20%). The absorbance was read at 557 nm and compared with a standard L-hydroxyproline.

Hexosamine content was estimated according to the method of Elson and Morgan [27]. Briefly, the reaction mixture contained acid hydrolysate, acetylacetone (2% in 1 N sodium carbonate), ethanol (96%), and Ehrlich reagent (2.67% of p-dimethylamino benzaldehyde in ethanol and HCl (1:1)). The absorbance was read at 530 nm and compared with the standard glucosamine hydrochloride. The results were represented as mg/g of dry tissue.

##### Masson’s trichrome (MT) staining for collagen fibers

MT staining was performed using the standard kit method (AMH labs, Bangalore, India) for the visualization of premature collagen fibers (stained light blue) and mature collagen fibers (stained dark blue) on paraffin-embedded sections.

#### *In vivo* antioxidant assays

Wet wound tissue (100 mg) was homogenized in tris HCl buffer (ice-cold, 10 mL) and used to estimate the content of lipid peroxides. The homogenized solution was further centrifuged (10,000 g, 30 min). The supernatant obtained was used to determine the activity of enzyme superoxide dismutase.

##### LPO assay

The LPO content in the homogenate was analyzed as suggested by Trush et al. [28]. The extent of malondialdehyde-thiobarbituric acid (MDA-TBA) chromophore produced per minute was used to measure the content of lipid peroxidation. Results were represented as nmol of MDA-TBA produced /mg protein/minute.

##### Superoxide dismutase (SOD)

SOD activity in the supernatant was determined according to Kono et al. [29]. Superoxide radicals produced during photo-oxidation of hydroxylamine hydrochloride reduce nitro blue tetrazolium into blue formazan. The activity of SOD capable of scavenging these superoxide radicals was noted and expressed as International units/mg of protein.

##### Total protein estimation

Total protein in the sample was estimated as described by Classics Lowry et al. [30]. In this method, peptide nitrogen under alkaline conditions reacts with copper ions and subsequently reduces Folin–Ciocalteau phosphomolybdic phosphotungstic acid to molybdenum blue. Optical density was read at 620 nm.

### Statistical analysis

Normality of the data and homogeneity of variance were checked using Shapiro Wilk test and Levene’s test respectively. Data were represented as mean ± SD and analyzed by ANOVA (one-way) followed by a posthoc test (Tukey’s test) to compare the groups. P-value ≤ 0.05 was considered statistically significant.

## Results

### Physical appearance and solubility

The obtained phosphorylated chitosan was brown in color. Phosphorylation resulted in depolymerization of the chitosan chain, making it less viscous and more water-soluble. These properties were prerequisites for a compound to easily degrade and provide numerous possible interactions with other biomolecules in the wound area. The solubility results of PC in three solvents were provided in Table 1.

**Table 1:** PC solubility in various solvents

Solvent	Solubility of PC (%)
Acetic acid	99.88 $\pm 0.028$
Sodium hydrogen carbonate	99.45 $\pm 0.180$
Distilled water	99.50 $\pm 0.173$

PC exhibited good solubility in water. Data expressed as mean $\pm \mathrm{SD}$

### Characterization of PC

The detailed elemental composition of phosphorylated chitosan and chitosan is depicted in the Table 2. The main elements detected are carbon, nitrogen, oxygen and phosphorous. The phosphorous content of PC and degree of substitution was found to be 6.09 $\pm 0.02$% and 0.39 $\pm 0.001$ respectively. Surface morphology and representative elemental composition of PC is provided in the supplementary data.

**Table 2:** Elemental composition of phosphorylated chitosan and DS_p_ (degree of substitution)

Sample	C (%)	N (%)	O (%)	P (%)	DS_p_
Chitosan	$57.60\pm$ 1.00	$5.68\pm 0.08$	$36.71\pm 1.08$	-	-
PC	$48.93\pm$ 1.20	$10.55\pm 0.62$	$34.42\pm 0.56$	$6.09\pm 0.02$	$0.39\pm 0.00$

Results presented as Mean ± SD (*n* = 3)

**Fig. 1:**
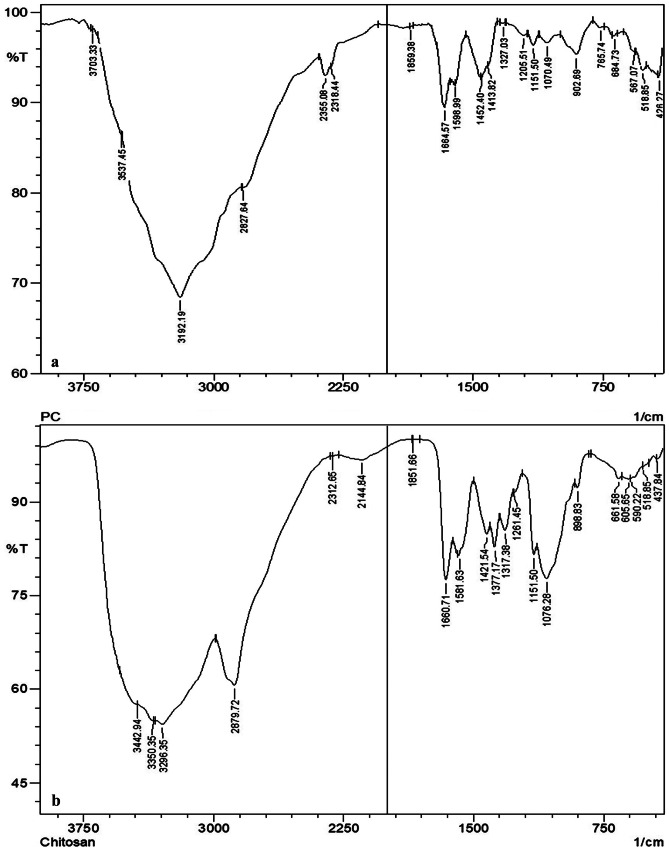
FTIR spectra of PC: (**a**, **b**) FTIR spectra of (**a**) PC, (**b**) Chitosan

In the FT-IR spectrum of phosphorylated chitosan (Fig. 1a) O–H stretching peak appeared at 3192 cm^−1^. The stretching peak for glycosidic bond C–O–C was noted at 1070 cm^−1^. The spectral peaks for amide I (C = O), and amide II (N–H bending) were noted at 1664 cm^−1^, and 1598 cm^−1^, respectively. The absorbance peak for P = O asymmetric stretching appeared at 1205 cm^−1^. The peak for C-O-P stretching of phosphate esters appeared at 902 cm^−1^. The spectral peak responsible for hydroxyl groups decreased in intensity and appeared at 3192 cm^−1^ compared to the peak at 3296 cm^−1^ in unmodified chitosan (Fig. 1b). These FTIR results suggested that phosphorylation primarily occurred at -OH groups of chitosan, which is in good agreement with previous reports [9, 15, 31].

**Fig. 2:**
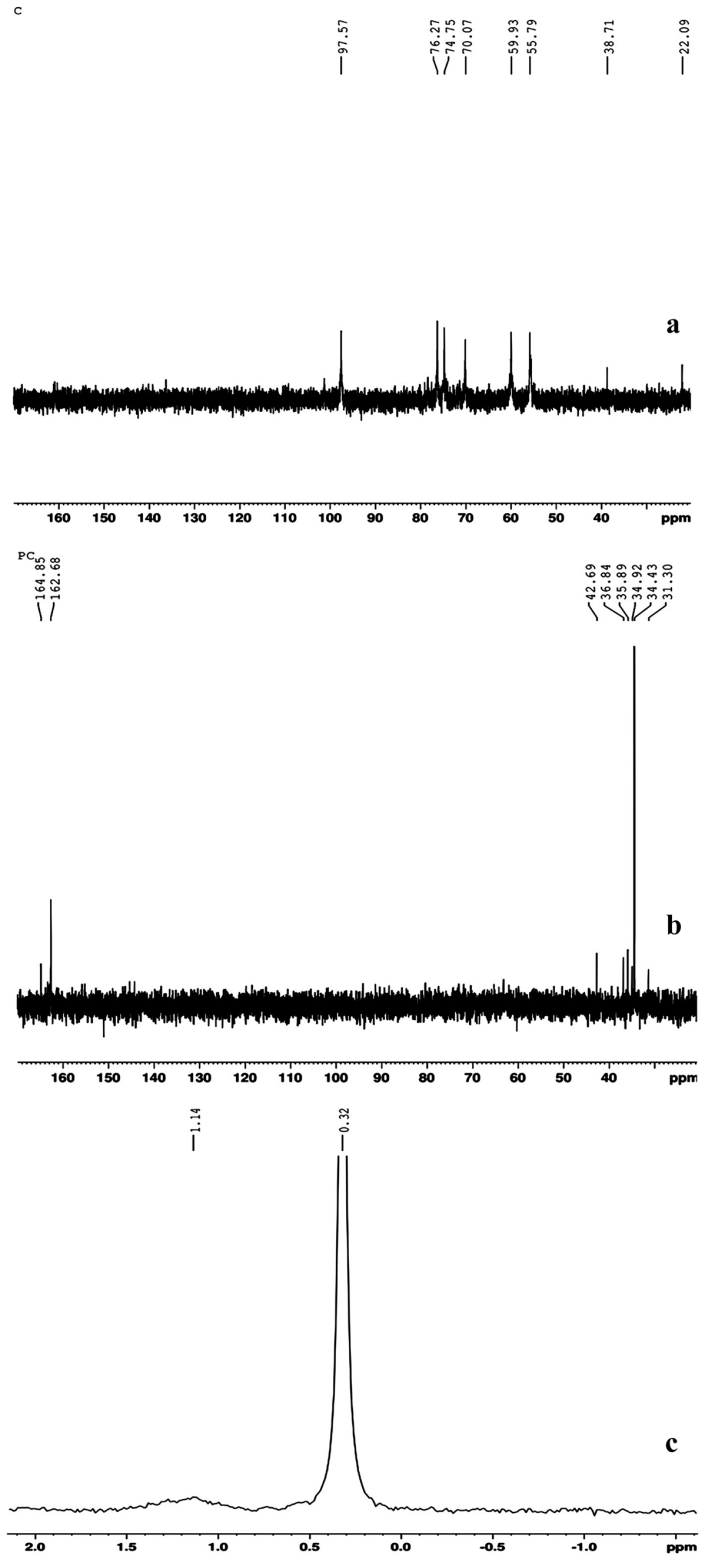
NMR spectra of PC: (**a**) ^13^C NMR spectra of Chitosan (**b**) ^13^C NMR spectra of PC (**c**) ^31^P NMR spectra of PC

The ^13^C NMR spectra of PC is presented in the Fig. 2. The chemical shifts of C-13 NMR of chitosan were δ = 97.57 ppm (C1), 76.27 ppm (C4), 74.75 ppm (C5), 70.07 ppm (C3), 59.93 ppm (C6), 55.79 ppm (C2), 38.71 ppm (Carbon of RCH-NH_2_) and 22.09 ppm (CH_3_CO) (Fig. 2a). For the ^13^C-NMR of phosphorylated chitosan, the new peak at δ = 164.85 and 162.68 ppm were attributed to the carbonyl groups of esters (Fig. 2b). In addition, the ^31^P-NMR spectrum of PC with chemical shift at δ = 0.32 ppm indicated the presence of phosphate (Fig. 2c). Overall, characterization studies of PC revealed the presence of phosphate groups at C-6 position of glucosamine and N-acetyl glucosamine respectively.

The final preparation of PC was free from any contamination of chitosan as indicated by thin layer chromatography (Supplementary data). PC had a number average molecular weight (Mn) of 78,921 g/mol as determined by osmotic pressure measurements.

### PC exhibited good antioxidant potential

**Fig. 3:**
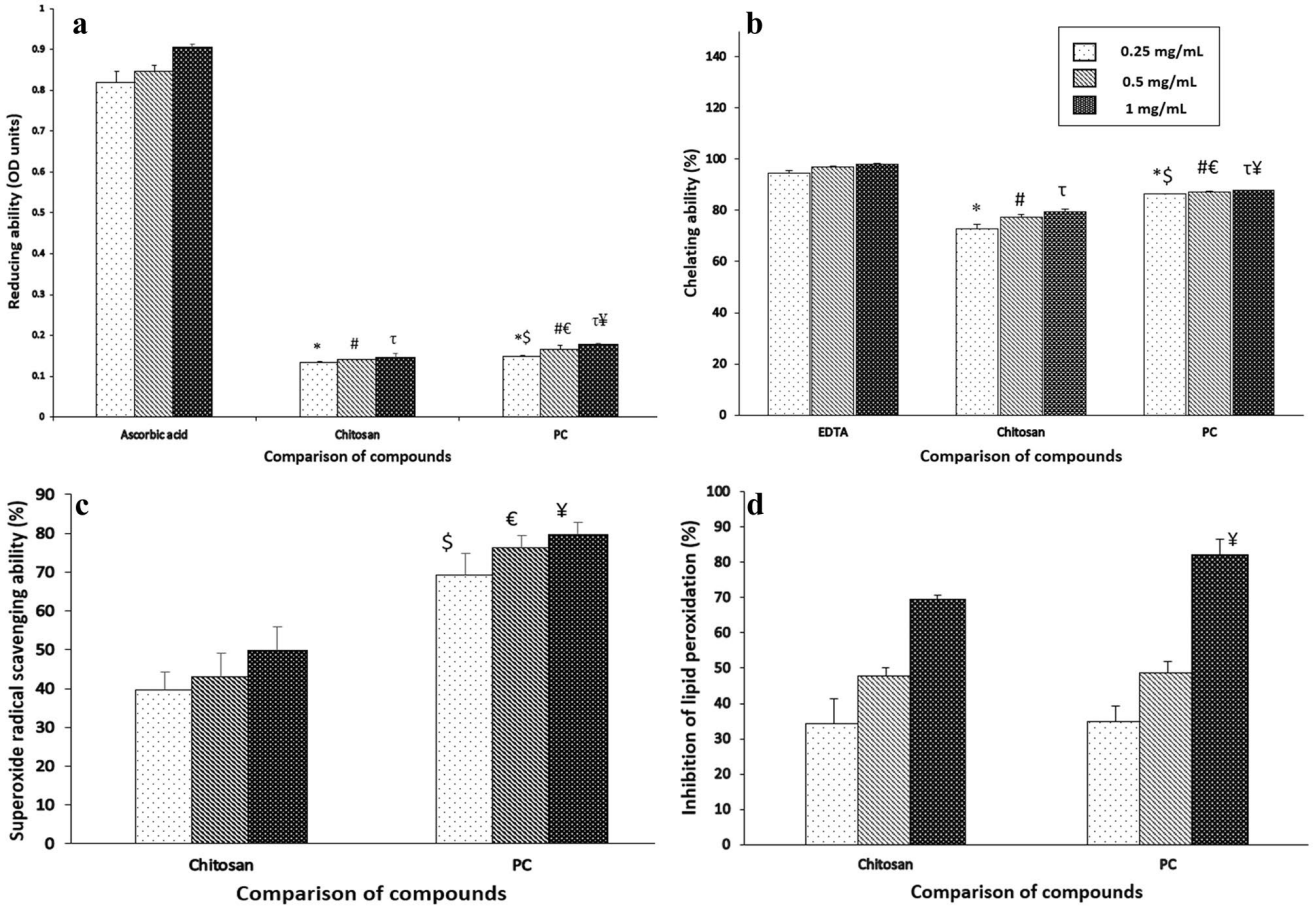
*In vitro* antioxidant ability of PC: (**a**) Reducing power of PC compared with ascorbic acid and chitosan (**b**) Chelating ability of PC compared with EDTA and chitosan (**c**) Superoxide scavenging activity of PC compared with chitosan. (**d**) Lipid peroxidation inhibiting ability of PC compared with chitosan. Results were expressed as mean ± SD (*n* = 6). *, ^#^ and τ signifies *p* ≤ 0.05 as compared to 0.25, 0.5 and 1 mg/ mL positive control (ascorbic acid or EDTA) and ^$, €^ and ^¥^, signifies *p* ≤ 0.05 as compared to 0.25, 0.5 and 1 mg/ mL chitosan, respectively

The reducing capacity of PC was higher compared to chitosan. PC demonstrated a concentration-dependent increase in reducing capacity. The reducing capacity of PC was significantly (*p* ≤ 0.05) higher at all tested concentrations as compared to the chitosan. Similarly, PC demonstrated significantly (*p* ≤ 0.05) improved metal chelating ability as compared to chitosan. The metal chelating ability of PC was found to be 1.19, 1.13, and 1.10 folds higher compared to chitosan at 0.25, 0.5, and 1 mg/mL, respectively. PC demonstrated good superoxide radical scavenging activity at all tested concentrations. The superoxide scavenging activity was 1.74, 1.77, and 1.60-folds at 0.25, 0.5, and 1 mg/mL as compared to chitosan. These values were statistically significantly (*p* ≤ 0.05) higher as compared to chitosan. The inhibition of lipid peroxidation by PC was significantly (*p* ≤ 0.05) higher (1.18 folds) at a concentration of 1 mg/mL as compared to the chitosan. However, no significant change in inhibition of lipid peroxidation was observed at lower PC concentrations as compared to chitosan. Overall, our results suggested that PC can be an effective antioxidant agent with improved antioxidant potential as compared to chitosan (Fig. 3).

### PC promoted good transitions through all the phases of wound healing

**Fig. 4:**
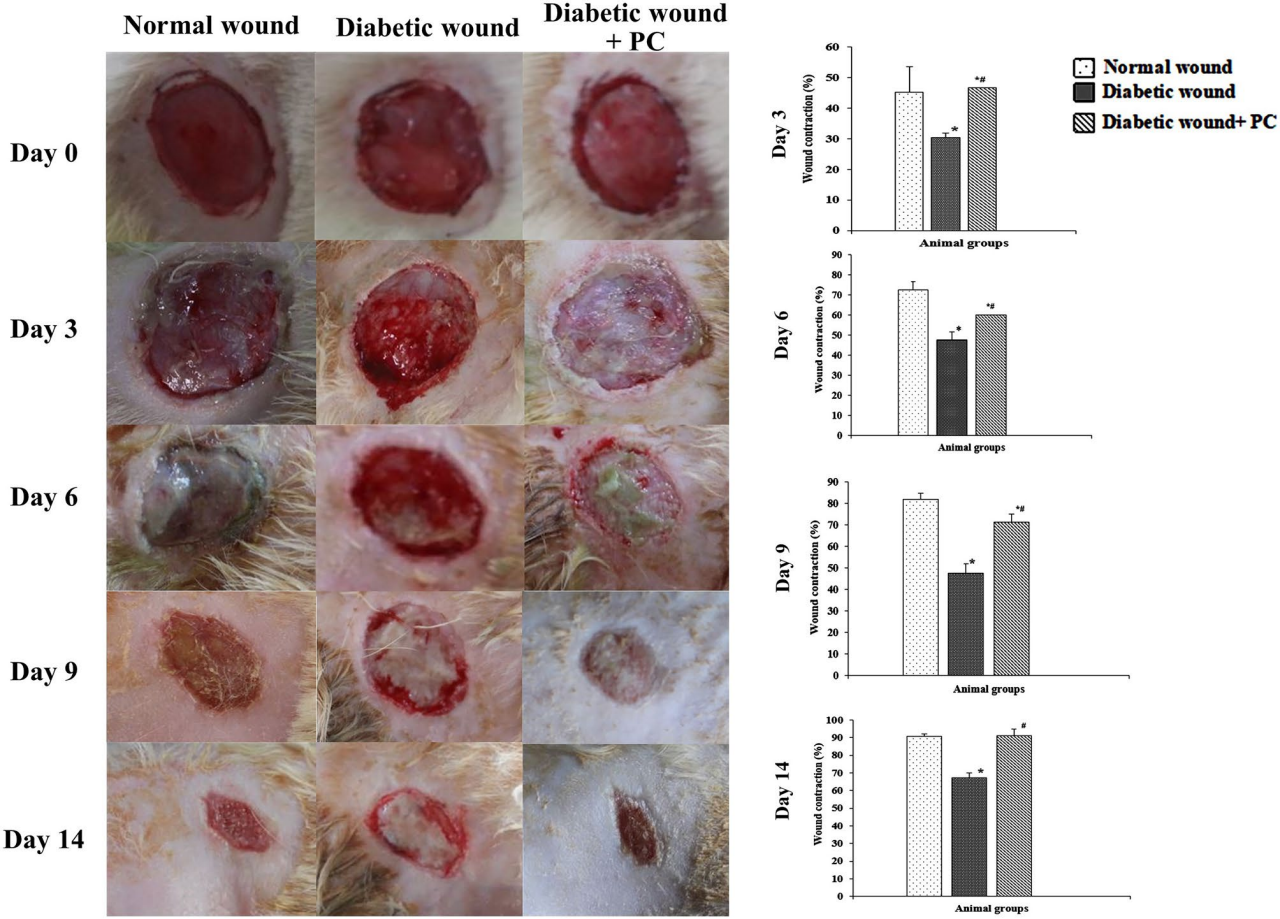
Wound area contracting ability of PC: Representative images of wounds and % wound contraction in normal wound, diabetic wound and diabetic wound + PC groups on day 0, 3, 6, 9 and 14 post surgery. Data were analysed using one-way ANOVA followed by post hoc test (Tukey’s test). *: represents *p* ≤ 0.05 when compared with normal wound and ^#^: represents *p* ≤ 0.05 when compared with diabetic wound

A gross morphological study of the wound revealed blood accumulation on day 0 in all groups depicting the hemostatic phase of wound healing. On day 3, swelling and inflammation were observed in diabetic wound + PC group similar to the normal wound group, whereas the diabetic wound group still demonstrated hemostatic phase. Day 9 evidenced drying of blood and initiation of scab formation in the diabetic wound + PC group and normal wound group indicating the proliferation phase, while the diabetic wound group still demonstrated the inflammatory phase. Further, day 14 of the study revealed higher wound contraction with scar formation in diabetic wound + PC treated group and normal wound group, indicating remodeling phase of wound healing. However, in diabetic wounds moderate inflammation with the initiation of scab formation was observed on day 14, indicating the overlapping inflammatory and proliferative phase (Fig. 4). Careful observation of the wound healing for 14 days showed a significant increase (*p* ≤ 0.05) in wound contraction in diabetic wound + PC group as compared to the diabetic wound group (Fig. 4). On day 3 of the study, PC-treated diabetic wounds showed a significantly (*p* ≤ 0.05) higher wound contraction (46.66%) as compared to the diabetic wounds (30.35%). Further on day 6, PC treated diabetic wound contraction was significantly higher (60%) compared to diabetic wound (47.62%). On day 9 and day 14 diabetic wound + PC treated group showed significantly higher (*p* ≤ 0.05) wound contraction (71%) and (91.11%) respectively as compared to (48%) and (67.26%) in diabetic wound group on the respective days.

### PC improved tissue morphology and collagen deposition

**Fig. 5:**
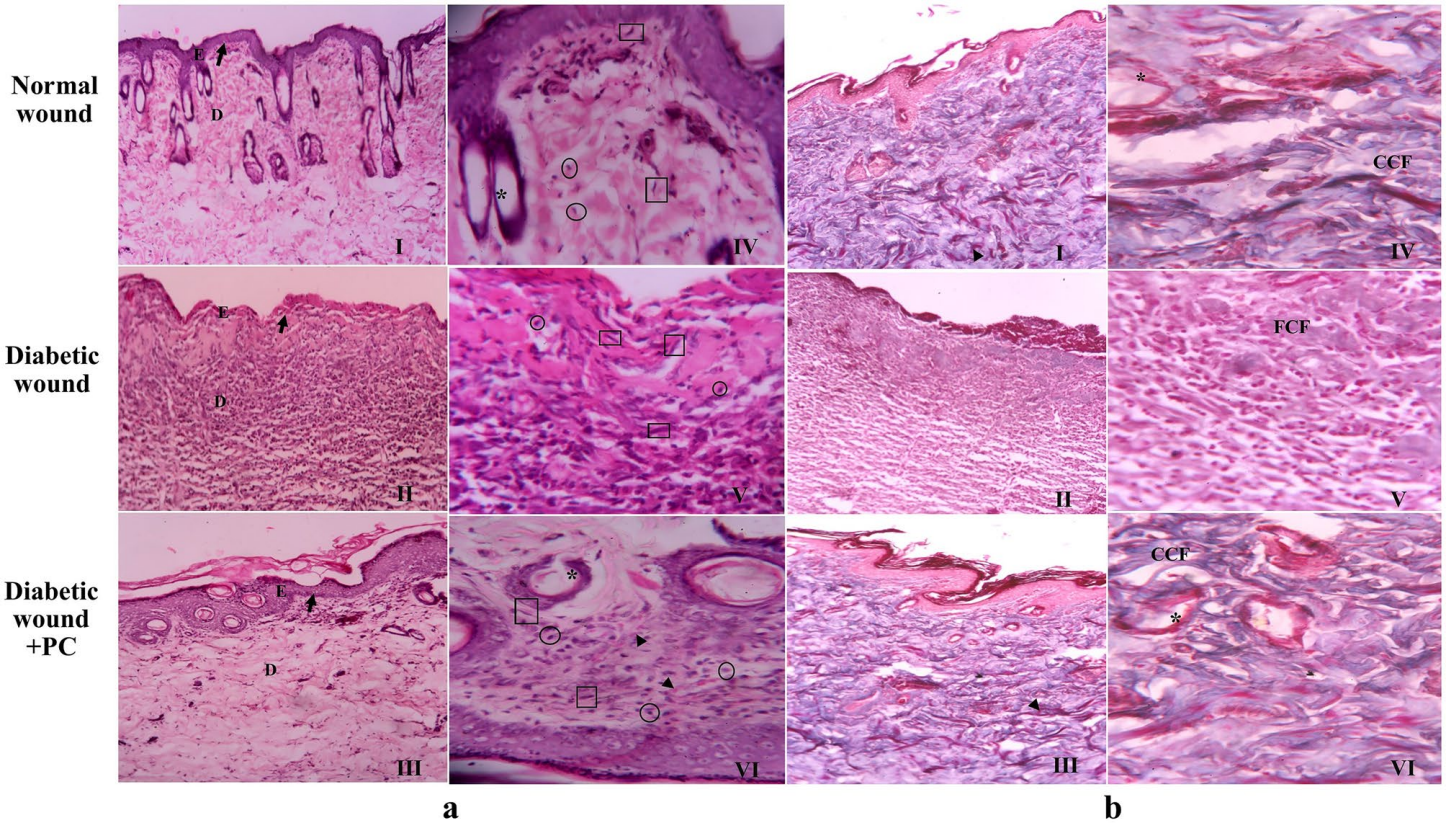
Histopathology of granulation tissue on day 14 of wound healing. (**a**) Photomicrographs of H & E-stained wounded skin tissue: I, II, III (100X magnification) and IV, V, VI (400X magnification). Diabetic wound + PC group showed thick epithelial layer, large number of fibroblasts, abundant blood capillaries compared to diabetic wound. E: Epidermis, D: Dermis, Dermal-epithelial junction- black arrow, Black arrowhead- neovascularisation, square- fibroblast, circle- macrophages, asterisk- neogenesis of skin appendages. (**b**) Photomicrograph of Masson’s trichrome stained wounded skin tissue: I, II, III (100X magnification) and IV, V, VI (400X magnification). Diabetic wound + PC group displayed enhanced collagen synthesis and maturation, and angiogenesis compared to untreated diabetic wound on day 14. CCF: Coarse Collagen Fibers, FFC: Fine Collagen Fibers, Black arrowhead: neovascularisation, Blue arrowhead-neogenesis of skin appendages

The tissue wound morphology on the 14^th^ day of wound healing was shown in Fig. 5a. The diabetic wounds showed the formation of a thin epithelial layer and infiltration of neutrophils. Additionally, fewer fibroblasts and blood vessels were also observed. However, diabetic wound + PC group demonstrated the formation of a thick epithelial layer, with abundant blood vessels and a good number of fibroblasts.

MT staining revealed the presence of collagen deposition and maturation in diabetic wound + PC group. The presence of large number of capillaries was also indicative of an improved angiogenic process in PC-treated wounds (Fig. 5b).

### PC elevated pro-healing collagen parameters and antioxidant defense status of the wounds

PC treatment showed significant (*p* ≤ 0.05) improvement in hydroxyproline (57.64%) and hexosamine (25.43%) as compared to the untreated diabetic wounds. PC-treated diabetic wounds also exhibited a significant increase in the activity of SOD (70.7%) and a significant decrease in the lipid peroxides (59%) as compared to the untreated diabetic wounds (Table 3).

**Table 3:** Status of hydroxyproline, hexosamine, superoxide dismutase and lipid peroxidation in granulation tissue after 14 days of PC treatment

**Parameters**	**Normal control**	**Diabetes control**	**PC + Diabetes**
**Hydroxyproline (mg/g)**	114.41 $\pm$ 4.79	43.21 $\pm$ 0.70	102.02 $\pm$ 0.89*^a^
**Hexosamine (mg/g)**	18.66 $\pm$ 0.65	12.78 $\pm$ 0.61	16.03 $\pm$ 0.49^a^
**Superoxide dismutase (IU/mg protein)**	1.52 $\pm$ 0.05	1.13 $\pm$ 0.09	1.93 $\pm$ 0.07*^a^
**Lipid peroxidation (nmol/mg protein)**	1.67 $\pm$ 0.37	8.49 $\pm$ 1.38	3.48 $\pm$ 0.26^a^

Data were analysed using one-way ANOVA followed by post hoc test (Tukey’s test). *: represents *p* ≤ 0.05 when compared with normal control and ^a^: represents *p* ≤ 0.05 when compared with diabetic control

## Discussion

The delayed wound healing in the case of diabetes is generally attributed to the hyperglycemia-induced generation of AGEs and AGE-related secondary complications [7]. Therefore, any compound which specifically inhibits the formation of AGEs or counteracts the complications related to AGEs may prove beneficial in accelerating wound healing.

In the present study, we synthesized phosphorylated chitosan and evaluated its wound healing potential in streptozotocin-induced diabetic rat model. The excisional wound was created, and wound healing effect of PC was evaluated every 3 days for 14 days. On day 3 of the study PC-treated diabetic wounds displayed swelling and inflammation with infiltration of large number of immune cells, which indicated the initiation of the inflammatory phase. Thus far no clot formation and no signs of inflammation in the diabetic wounds indicated a defective initiation phase of wound healing [32]. The progression of PC-treated diabetic wounds towards a full-fledged inflammatory phase was noted by the 6^th^ day whereas, untreated diabetic wounds were still in the low-grade inflammatory phase. Further, on day 9, PC-treated diabetic wounds demonstrated drying of the blood and formation of scabs, which were typical characteristics of endothelial and epithelial cell proliferation [33]. Day 14 of the study noted formation of scar tissue and re-epithelization of the wound area in the PC-treated diabetic and normal wounds, which indicated the initiation of the remodeling phase [34]. However, a low-grade inflammatory phase and early remodeling phase were noted in the untreated diabetic wounds till the 14^th^ day of the study. These results demonstrated that PC accelerated the delayed wound healing processes in diabetes.

Hyperglycemic conditions in diabetes mellitus induce the generation of AGEs in wound tissues via the Maillard reaction wherein, carboxyl group of glucose reacts with amino groups of Lys and Arg residues of proteins involved in various phases of wound healing rendering them inactive [35]. The amine functional groups of PC exerted competitive inhibitory effect and competed with Lys and Arg residues of key proteins for the carboxyl group of glucose and hence reducing the formation of AGEs. Similar inhibitory effects of chitosan derivatives on AGEs are also noted by other researchers [36, 37]. Additionally, the amine functional groups of PC can electrostatically interact with the negative cell membranes and immobilize the cells in the wound area [38]. These interactions agglutinate the RBCs and activate the platelets required for the initial plug formation in the wound area [39]. Furthermore, the presence of phosphate group in PC mimics polyphosphates (PolyPs), an anionic natural substance released from activated platelets. PolyPs bind to a variety of proteins like factor XII, factor V, factor XI, prekallikrein, and kininogen. These interactions results in facilitation of the blood coagulation cascade and further promote thrombin generation and clot formation [10, 38].

During the inflammatory phase of wound healing, reactive oxygen species play a major role in the orchestration of inflammatory response [40]. They act as second messengers to immunocytes and also bring about the respiratory burst (increase in ROS) leading to the destruction of pathogens [41]. However, excessive ROS production leads to oxidative stress-related tissue damage and impaired wound healing. Several studies reported the negative influence of oxidative stress on non-healing diabetic wounds [42–44]. Therefore, a precise balance of ROS is critical in effective wound healing [45]. PC with active functional groups at C-2, C-3, and C-6 positions can be useful in counteracting this oxidative stress through various mechanisms [41]. The hydroxyl groups at C-3 and C-6 positions of PC lead to H abstraction reactions with •OH (hydroxyl radical) [46, 47]. Active amino groups at C-2 position react with the protons from the surrounding medium and scavenge free radicals through addition reactions [48].

The phosphate group improved antioxidant and metal chelating properties of chitosan as observed in *in vitro* and *in vivo* antioxidant studies. Reducing ability is an important parameter to ascertain the antioxidant activity of any compound [49]. Phosphorylated compounds with “HOPO_2_H_2_ groups” can act as reducing agents or reductones [50]. Reductones can easily donate hydrogen atoms to O_2_•^−^ (Superoxide radical) generated in the wound area, thus finally converting them into stable products [51]. Increased oxidative stress in the wound area also releases ferrous iron (Fe^2+^) due to rupturing of the RBCs and fibroblasts [52]. These free Fe^2+^can undergo Fenton’s reaction wherein Fe^2+^ is oxidized by H_2_O_2_ to give ferric iron (Fe^3+^), which generates more toxic hydroxyl radicals. These hydroxyl radicals further increase oxidative stress by the peroxidation of lipids [53]. PC with good ferrous iron chelating ability can limit the participation of Fe^2+^ in Fenton’s reaction and thereby reduce further oxidative stress [54]. A decrease in granulation tissue LPO also correlated with these findings [55].

In the late proliferative phase and early remodeling phase, PC played an important role which is reflected in increased collagen deposition, angiogenesis, epidermal proliferation, and an increase in the number of fibroblasts. In these phases, PC can be advantageous in two ways. Firstly, the low molecular weight PC once placed on the wound, adheres to the fibroblasts, keratinocytes, and endothelial cells through electrostatic interactions and favors smooth migration and proliferation [10]. Secondly, the amphoteric nature of PC can also immobilize the growth factors such as FGF-2, VEGF, EGF, etc. around these cells, which can further stimulate the growth of these cells [11, 56]. Dynamic interactions between the growth factors and the ECM are also integral to the effective healing of wounds. Furthermore, the glucosamine units of PC structurally resemble the amino sugars of glycosaminoglycans (GAGs) which are required for the formation of ECM. PC on hydrolysis releases glucosamine units which can be incorporated during GAG synthesis [10, 12]. These GAGs of ECM can further serve to sequester and protect the growth factors from degradation and enhanced their activity around cells. Overall, these properties of PC may bring about good interactions of fibroblasts, endothelial cells and keratinocytes with their respective growth factors ultimately leading to efficient collagen deposition, angiogenesis and epidermal regeneration.

## Conclusion

The results of the present study demonstrated that PC accelerated the process of wound healing in diabetic rats. Considering this, PC can be further used in the treatment of diabetic foot ulcers. However, before its clinical application, absolute prospective clinical trials to examine its safety, effectiveness, and possible action in humans are recommended.

## Supplementary Information

Below is the link to the electronic supplementary material.

Supplementary file1 (DOCX 590 KB)

## Data Availability

The datasets generated during and/or analysed during the current study are available from the corresponding author on reasonable request.
