# Supplementary material for: Phosphorylated chitosan accelerates dermal wound healing in diabetic wistar rats
Source: Glycoconj J. 2022 Nov 30;40(1):19–31. doi: 10.1007/s10719-022-10093-5 (PMC9925528; doi:10.1007/s10719-022-10093-5)
Supplement: Supplementary file 1 — Supplementary file1 (DOCX 590 KB) [file 10719_2022_10093_MOESM1_ESM.docx]

**Supplementary data:**

1. **Scanning electron microscopy and elemental analysis (SEM-EDX) of Chitosan and PC**


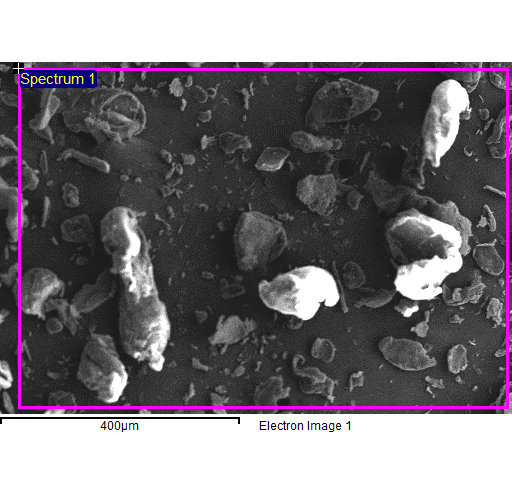


**Supplementary figure 1:** Surface morphology and elemental composition of chitosan


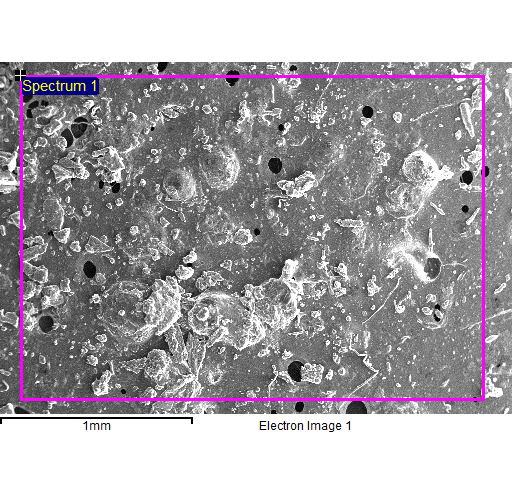


**Supplementary figure 2:** Surface morphology and elemental composition of PC

**Determination of degree of substitution:**

The degree of substitution (DS_p_) was determined using the formula (Deng et al., 2015):

$$DSp=\frac{162\times p\%}{(31\times100)-(96\times p\%)}$$

Where,

P% is phosphorous content

162 is the relative molecular weight of the unmodified chitosan monomer

31 is the mass of phosphorous atom

1. s the added molecular weight when -OH was substituted by -OPO_3_H_2_
2. **Thin layer chromatography for the determination of purity of PC**


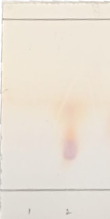


**C**

**PC**

**Supplementary figure 3:Thin layer chromatography analysis of PC.** C: chitosan and PC: phosphorylated chitosan

**References:**

Deng C, Fu H, Xu J, Shang J, Cheng Y. Physiochemical and biological properties of phosphorylated polysaccharides from Dictyophora indusiata. International journal of biological macromolecules. 2015 Jan 1;72:894-9.
